# Supplementary material for: An N-terminal motif in NLR immune receptors is functionally conserved across distantly related plant species
Source: eLife. 2019 Nov 27;8:e49956. doi: 10.7554/eLife.49956 (PMC6944444; doi:10.7554/eLife.49956)
Supplement: Supplementary file 1. [file elife-49956-supp1.pptx]

## Slide 1
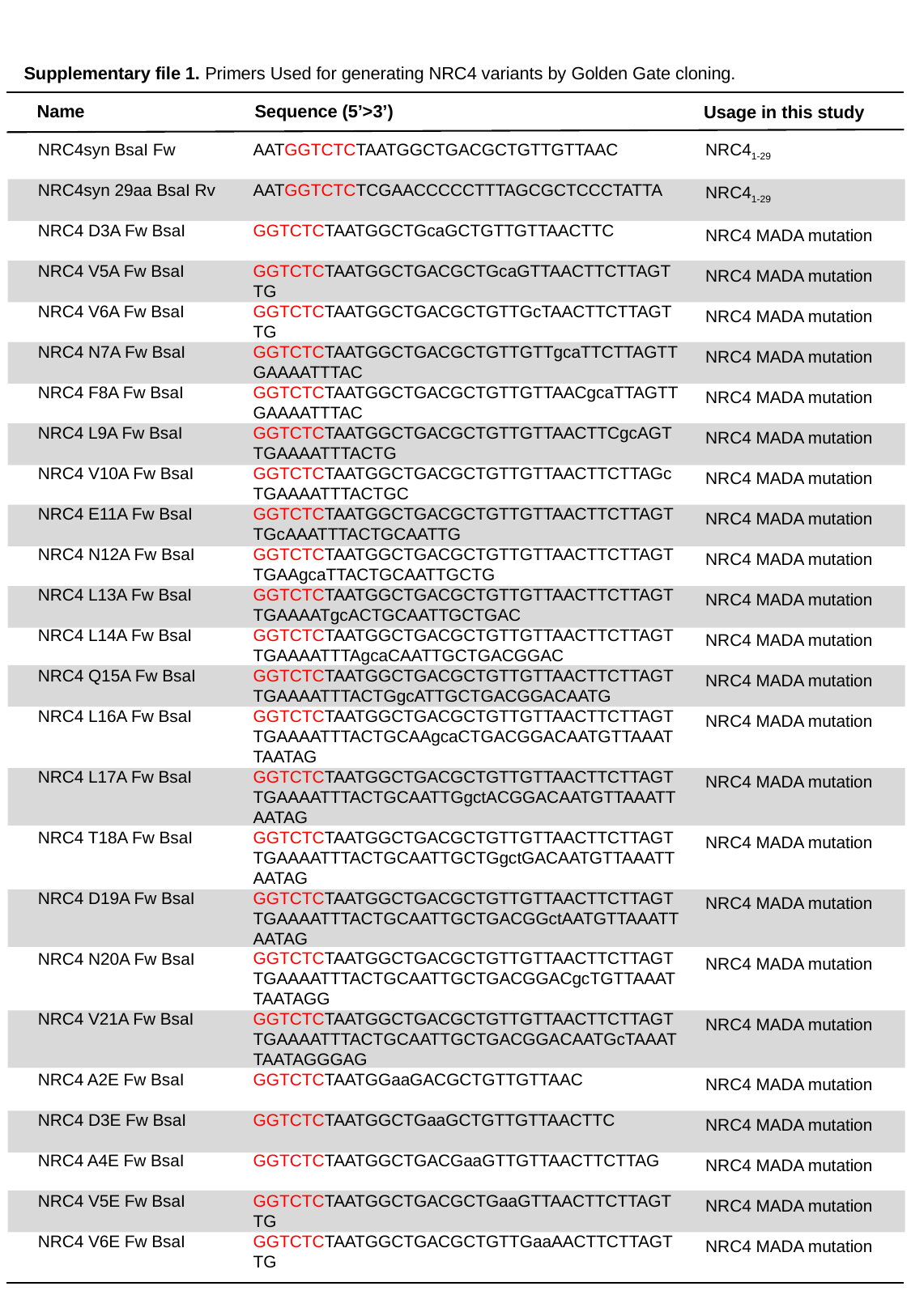

Supplementary file 1. Primers Used for generating NRC4 variants by Golden Gate cloning.
Name
Sequence (5’>3’)
Usage in this study
NRC41-29
NRC41-29
NRC4 MADA mutation
NRC4 MADA mutation
NRC4 MADA mutation
NRC4 MADA mutation
NRC4 MADA mutation
NRC4 MADA mutation
NRC4 MADA mutation
NRC4 MADA mutation
NRC4 MADA mutation
NRC4 MADA mutation
NRC4 MADA mutation
NRC4 MADA mutation
NRC4 MADA mutation
NRC4 MADA mutation
NRC4 MADA mutation
NRC4 MADA mutation
NRC4 MADA mutation
NRC4 MADA mutation
NRC4 MADA mutation
NRC4 MADA mutation
NRC4 MADA mutation
NRC4 MADA mutation
NRC4 MADA mutation
NRC4syn BsaI Fw
NRC4syn 29aa BsaI Rv
NRC4 D3A Fw BsaI
NRC4 V5A Fw BsaI
NRC4 V6A Fw BsaI
NRC4 N7A Fw BsaI
NRC4 F8A Fw BsaI
NRC4 L9A Fw BsaI
NRC4 V10A Fw BsaI
NRC4 E11A Fw BsaI
NRC4 N12A Fw BsaI
NRC4 L13A Fw BsaI
NRC4 L14A Fw BsaI
NRC4 Q15A Fw BsaI
NRC4 L16A Fw BsaI
NRC4 L17A Fw BsaI
NRC4 T18A Fw BsaI
NRC4 D19A Fw BsaI
NRC4 N20A Fw BsaI
NRC4 V21A Fw BsaI
NRC4 A2E Fw BsaI
NRC4 D3E Fw BsaI
NRC4 A4E Fw BsaI
NRC4 V5E Fw BsaI
NRC4 V6E Fw BsaI
AATGGTCTCTAATGGCTGACGCTGTTGTTAAC
AATGGTCTCTCGAACCCCCTTTAGCGCTCCCTATTA
GGTCTCTAATGGCTGcaGCTGTTGTTAACTTC
GGTCTCTAATGGCTGACGCTGcaGTTAACTTCTTAGTTG
GGTCTCTAATGGCTGACGCTGTTGcTAACTTCTTAGTTG
GGTCTCTAATGGCTGACGCTGTTGTTgcaTTCTTAGTTGAAAATTTAC
GGTCTCTAATGGCTGACGCTGTTGTTAACgcaTTAGTTGAAAATTTAC
GGTCTCTAATGGCTGACGCTGTTGTTAACTTCgcAGTTGAAAATTTACTG
GGTCTCTAATGGCTGACGCTGTTGTTAACTTCTTAGcTGAAAATTTACTGC
GGTCTCTAATGGCTGACGCTGTTGTTAACTTCTTAGTTGcAAATTTACTGCAATTG
GGTCTCTAATGGCTGACGCTGTTGTTAACTTCTTAGTTGAAgcaTTACTGCAATTGCTG
GGTCTCTAATGGCTGACGCTGTTGTTAACTTCTTAGTTGAAAATgcACTGCAATTGCTGAC
GGTCTCTAATGGCTGACGCTGTTGTTAACTTCTTAGTTGAAAATTTAgcaCAATTGCTGACGGAC
GGTCTCTAATGGCTGACGCTGTTGTTAACTTCTTAGTTGAAAATTTACTGgcATTGCTGACGGACAATG
GGTCTCTAATGGCTGACGCTGTTGTTAACTTCTTAGTTGAAAATTTACTGCAAgcaCTGACGGACAATGTTAAATTAATAG
GGTCTCTAATGGCTGACGCTGTTGTTAACTTCTTAGTTGAAAATTTACTGCAATTGgctACGGACAATGTTAAATTAATAG
GGTCTCTAATGGCTGACGCTGTTGTTAACTTCTTAGTTGAAAATTTACTGCAATTGCTGgctGACAATGTTAAATTAATAG
GGTCTCTAATGGCTGACGCTGTTGTTAACTTCTTAGTTGAAAATTTACTGCAATTGCTGACGGctAATGTTAAATTAATAG
GGTCTCTAATGGCTGACGCTGTTGTTAACTTCTTAGTTGAAAATTTACTGCAATTGCTGACGGACgcTGTTAAATTAATAGG
GGTCTCTAATGGCTGACGCTGTTGTTAACTTCTTAGTTGAAAATTTACTGCAATTGCTGACGGACAATGcTAAATTAATAGGGAG
GGTCTCTAATGGaaGACGCTGTTGTTAAC
GGTCTCTAATGGCTGaaGCTGTTGTTAACTTC
GGTCTCTAATGGCTGACGaaGTTGTTAACTTCTTAG
GGTCTCTAATGGCTGACGCTGaaGTTAACTTCTTAGTTG
GGTCTCTAATGGCTGACGCTGTTGaaAACTTCTTAGTTG

## Slide 2
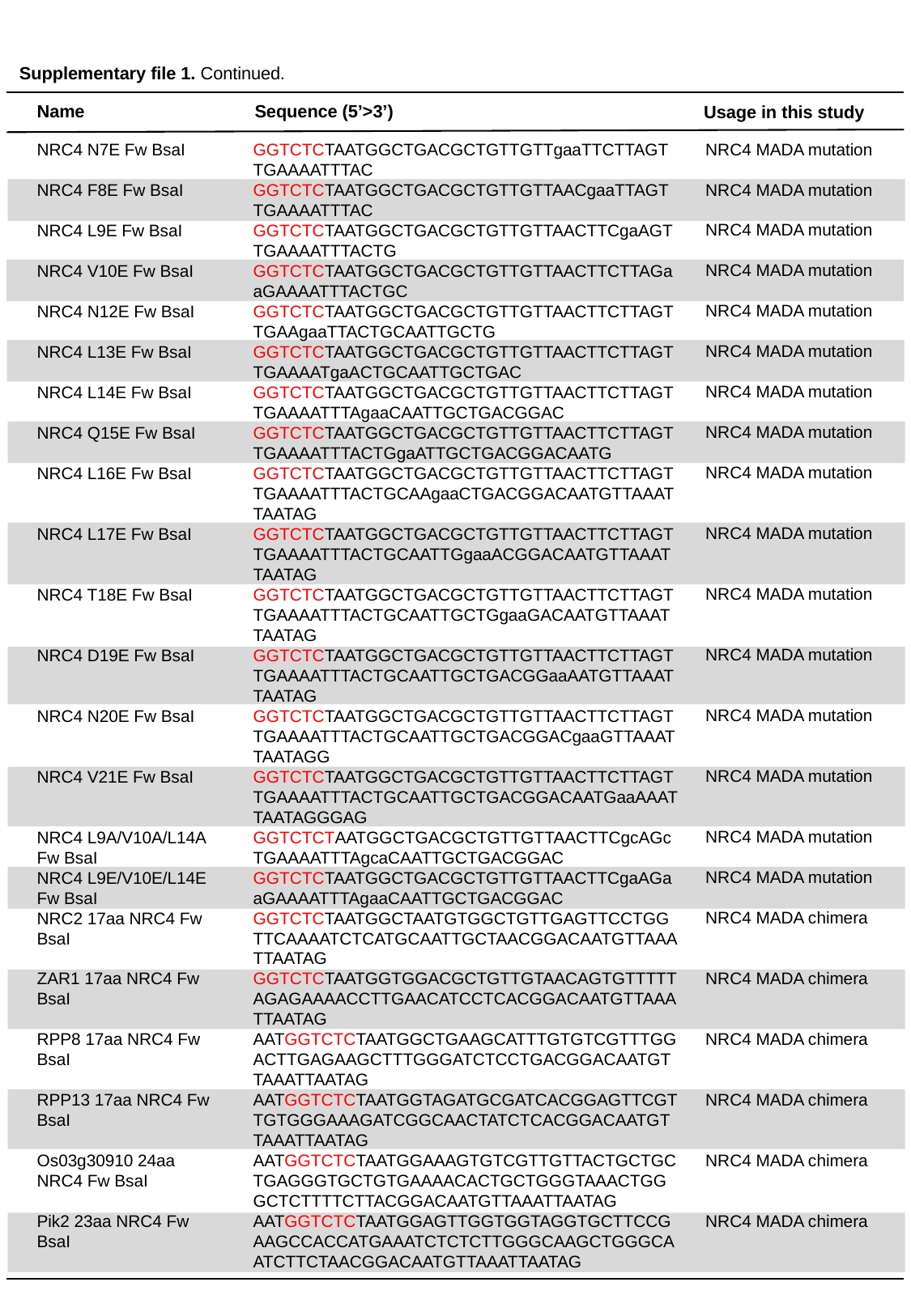

Supplementary file 1. Continued.
Name
Sequence (5’>3’)
Usage in this study
NRC4 MADA mutation
NRC4 MADA mutation
NRC4 MADA mutation
NRC4 MADA mutation
NRC4 MADA mutation
NRC4 MADA mutation
NRC4 MADA mutation
NRC4 MADA mutation
NRC4 MADA mutation
NRC4 MADA mutation
NRC4 MADA mutation
NRC4 MADA mutation
NRC4 MADA mutation
NRC4 MADA mutation
NRC4 MADA mutation
NRC4 MADA mutation
NRC4 MADA chimera
NRC4 MADA chimera
NRC4 MADA chimera
NRC4 MADA chimera
NRC4 MADA chimera
NRC4 MADA chimera
NRC4 N7E Fw BsaI
NRC4 F8E Fw BsaI
NRC4 L9E Fw BsaI
NRC4 V10E Fw BsaI
NRC4 N12E Fw BsaI
NRC4 L13E Fw BsaI
NRC4 L14E Fw BsaI
NRC4 Q15E Fw BsaI
NRC4 L16E Fw BsaI
NRC4 L17E Fw BsaI
NRC4 T18E Fw BsaI
NRC4 D19E Fw BsaI
NRC4 N20E Fw BsaI
NRC4 V21E Fw BsaI
NRC4 L9A/V10A/L14A Fw BsaI
NRC4 L9E/V10E/L14E Fw BsaI
NRC2 17aa NRC4 Fw BsaI
ZAR1 17aa NRC4 Fw BsaI
RPP8 17aa NRC4 Fw BsaI
RPP13 17aa NRC4 Fw BsaI
Os03g30910 24aa NRC4 Fw BsaI
Pik2 23aa NRC4 Fw BsaI
GGTCTCTAATGGCTGACGCTGTTGTTgaaTTCTTAGTTGAAAATTTAC
GGTCTCTAATGGCTGACGCTGTTGTTAACgaaTTAGTTGAAAATTTAC
GGTCTCTAATGGCTGACGCTGTTGTTAACTTCgaAGTTGAAAATTTACTG
GGTCTCTAATGGCTGACGCTGTTGTTAACTTCTTAGaaGAAAATTTACTGC
GGTCTCTAATGGCTGACGCTGTTGTTAACTTCTTAGTTGAAgaaTTACTGCAATTGCTG
GGTCTCTAATGGCTGACGCTGTTGTTAACTTCTTAGTTGAAAATgaACTGCAATTGCTGAC
GGTCTCTAATGGCTGACGCTGTTGTTAACTTCTTAGTTGAAAATTTAgaaCAATTGCTGACGGAC
GGTCTCTAATGGCTGACGCTGTTGTTAACTTCTTAGTTGAAAATTTACTGgaATTGCTGACGGACAATG
GGTCTCTAATGGCTGACGCTGTTGTTAACTTCTTAGTTGAAAATTTACTGCAAgaaCTGACGGACAATGTTAAATTAATAG
GGTCTCTAATGGCTGACGCTGTTGTTAACTTCTTAGTTGAAAATTTACTGCAATTGgaaACGGACAATGTTAAATTAATAG
GGTCTCTAATGGCTGACGCTGTTGTTAACTTCTTAGTTGAAAATTTACTGCAATTGCTGgaaGACAATGTTAAATTAATAG
GGTCTCTAATGGCTGACGCTGTTGTTAACTTCTTAGTTGAAAATTTACTGCAATTGCTGACGGaaAATGTTAAATTAATAG
GGTCTCTAATGGCTGACGCTGTTGTTAACTTCTTAGTTGAAAATTTACTGCAATTGCTGACGGACgaaGTTAAATTAATAGG
GGTCTCTAATGGCTGACGCTGTTGTTAACTTCTTAGTTGAAAATTTACTGCAATTGCTGACGGACAATGaaAAATTAATAGGGAG
GGTCTCTAATGGCTGACGCTGTTGTTAACTTCgcAGcTGAAAATTTAgcaCAATTGCTGACGGAC
GGTCTCTAATGGCTGACGCTGTTGTTAACTTCgaAGaaGAAAATTTAgaaCAATTGCTGACGGAC
GGTCTCTAATGGCTAATGTGGCTGTTGAGTTCCTGGTTCAAAATCTCATGCAATTGCTAACGGACAATGTTAAATTAATAG
GGTCTCTAATGGTGGACGCTGTTGTAACAGTGTTTTTAGAGAAAACCTTGAACATCCTCACGGACAATGTTAAATTAATAG
AATGGTCTCTAATGGCTGAAGCATTTGTGTCGTTTGGACTTGAGAAGCTTTGGGATCTCCTGACGGACAATGTTAAATTAATAG
AATGGTCTCTAATGGTAGATGCGATCACGGAGTTCGTTGTGGGAAAGATCGGCAACTATCTCACGGACAATGTTAAATTAATAG
AATGGTCTCTAATGGAAAGTGTCGTTGTTACTGCTGCTGAGGGTGCTGTGAAAACACTGCTGGGTAAACTGGGCTCTTTTCTTACGGACAATGTTAAATTAATAG
AATGGTCTCTAATGGAGTTGGTGGTAGGTGCTTCCGAAGCCACCATGAAATCTCTCTTGGGCAAGCTGGGCAATCTTCTAACGGACAATGTTAAATTAATAG

## Slide 3
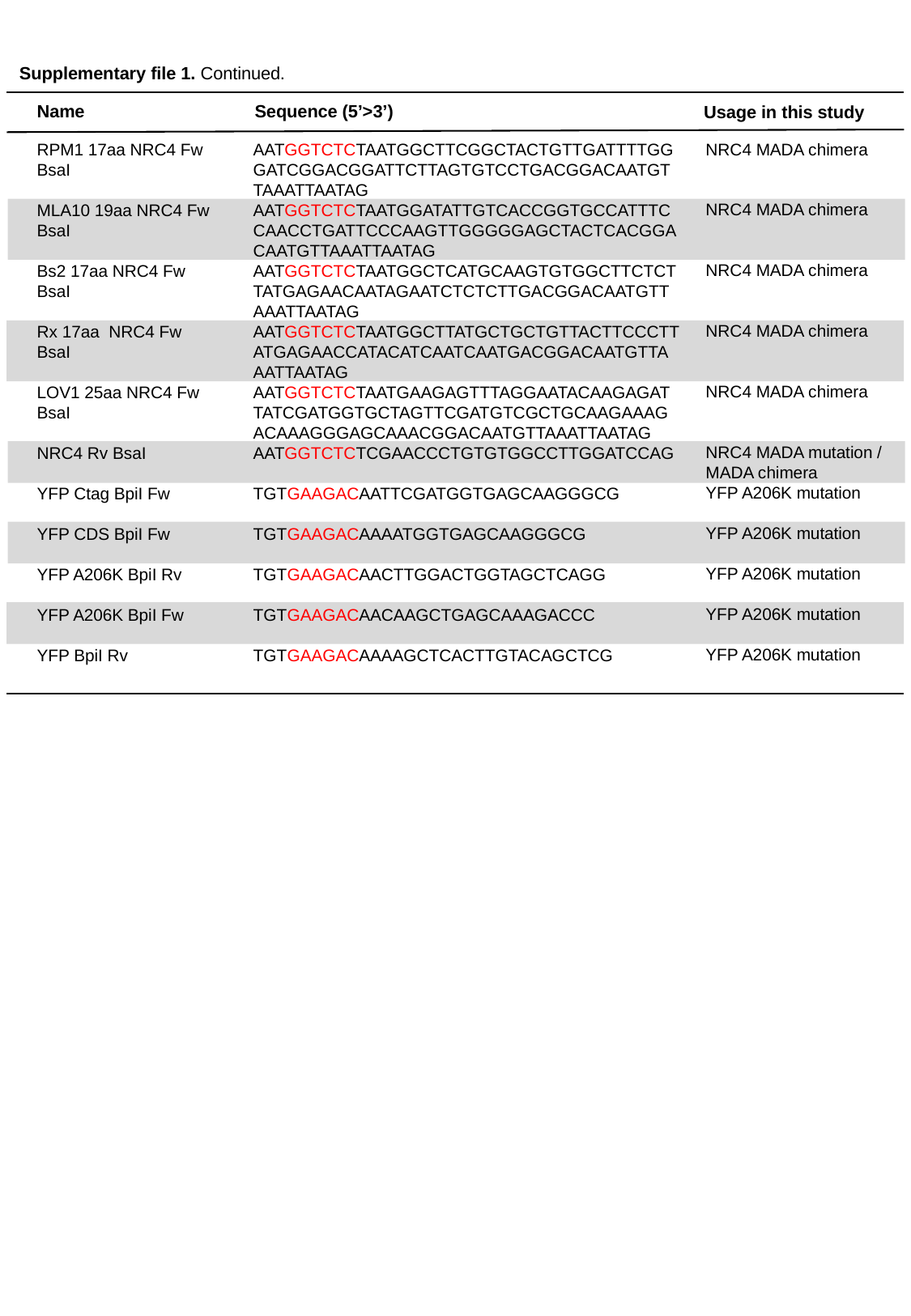

Supplementary file 1. Continued.
Name
Sequence (5’>3’)
Usage in this study
NRC4 MADA chimera
NRC4 MADA chimera
NRC4 MADA chimera
NRC4 MADA chimera
NRC4 MADA chimera
NRC4 MADA mutation /
MADA chimera
YFP A206K mutation
YFP A206K mutation
YFP A206K mutation
YFP A206K mutation
YFP A206K mutation
RPM1 17aa NRC4 Fw BsaI
MLA10 19aa NRC4 Fw BsaI
Bs2 17aa NRC4 Fw BsaI
Rx 17aa NRC4 Fw BsaI
LOV1 25aa NRC4 Fw BsaI
NRC4 Rv BsaI
YFP Ctag BpiI Fw
YFP CDS BpiI Fw
YFP A206K BpiI Rv
YFP A206K BpiI Fw
YFP BpiI Rv
AATGGTCTCTAATGGCTTCGGCTACTGTTGATTTTGGGATCGGACGGATTCTTAGTGTCCTGACGGACAATGTTAAATTAATAG
AATGGTCTCTAATGGATATTGTCACCGGTGCCATTTCCAACCTGATTCCCAAGTTGGGGGAGCTACTCACGGACAATGTTAAATTAATAG
AATGGTCTCTAATGGCTCATGCAAGTGTGGCTTCTCTTATGAGAACAATAGAATCTCTCTTGACGGACAATGTTAAATTAATAG
AATGGTCTCTAATGGCTTATGCTGCTGTTACTTCCCTTATGAGAACCATACATCAATCAATGACGGACAATGTTAAATTAATAG
AATGGTCTCTAATGAAGAGTTTAGGAATACAAGAGATTATCGATGGTGCTAGTTCGATGTCGCTGCAAGAAAGACAAAGGGAGCAAACGGACAATGTTAAATTAATAG
AATGGTCTCTCGAACCCTGTGTGGCCTTGGATCCAG
TGTGAAGACAATTCGATGGTGAGCAAGGGCG
TGTGAAGACAAAATGGTGAGCAAGGGCG
TGTGAAGACAACTTGGACTGGTAGCTCAGG
TGTGAAGACAACAAGCTGAGCAAAGACCC
TGTGAAGACAAAAGCTCACTTGTACAGCTCG
